# Public Highways

**Published:** 1893-02

**Authors:** 


### EDITORIAL.

---

### PUBLIC HIGHWAYS.

Practical experience has shown that good roads increase the number of valuable horses in a community, as they allow the horse to be used to an advantage both as a means of transport and as an article of luxury for pleasure driving. Increased numbers of valuable horses means a better and more lucrative practice for the veterinarian. The veterinarian, of all others, is the one therefore who should be interested in the improvement of roads, and he should make it his study to know how they can best be improved, and so become an adviser of his clients and those who have the making of them. In this view we publish in this number of the JOURNAL a clever article by Miss Sarah Cooper Hewitt, and we append a form of petition to Congress, blanks of which will be forwarded from this office to veterinarians desirous of obtaining signatures and aiding in the object:

"*To the Honorable Senate and House of Representatives in Congress assembled:*

"We, the undersigned, citizens of the United States, hereby most respectfully petition that there be founded in the City of Washington, in the District of Columbia, a Road Department, similar to the Agricultural Department, for the purpose of promoting knowledge in the art of constructing and maintaining roads; and we ask that in such department provision be made for teaching students so that they may become skilled road engineers.

"In connection with this Road Department we request that there be established a permanent Exhibit in which shall be shown sections of roads illustrating various methods of construction and also the best road materials and machinery.

"We further petition that Congress appropriate funds sufficient to erect a building at the World's Columbian Exposition for the purpose of a comprehensive road exhibit.

"NOTE.—When this sheet is filled with names, please attach other sheets and secure as many signatures as possible. When this is done, please forward it as soon as possible to the address given below. Copies of this petition have been sent to various parts of the country, and it is hoped that at least a million signatures will be secured. All petitions sent in before January 1st will be united in one great petition, which will be presented to Congress, and the result will be, no doubt, the establishment of a Road Department, an Institute of Road Engineering, and a permanent Road Exhibit in the City of Washington, and a comprehensive exhibit of the construction and maintenance of roads at the World's Columbian Exposition.

"P. O. Box B, Boston, Mass. ALBERT A. POPE."

